# Associations between mental health disorder and management of physical chronic conditions in China: a pooled cross-sectional analysis

**DOI:** 10.1038/s41598-021-85126-4

**Published:** 2021-03-11

**Authors:** Zheng Zhang, Grace Sum, Vicky Mengqi Qin, Yang Zhao, Tilahun Nigatu Haregu, Brian Oldenburg, John Tayu Lee

**Affiliations:** 1 School of Population and Global Health, University of Melbourne, Melbourne, VIC Australia; 2 Saw Swee Hock School of Public Health, National University of Singapore, Singapore, Singapore; 3 The George Institute for Global Health at Peking University Health Science Centre, Beijing, China; 4 WHO Collaborating Centre on Implementation Research for Prevention and Control of Noncommunicable Disease, Melbourne, VIC Australia; 5 The Nossal Institute for Global Health, The University of Melbourne, Melbourne, VIC Australia; 6 Department of Primary Care and Public Health, School of Public Health, Imperial College, London, UK

**Keywords:** Public health, Diagnosis

## Abstract

Physical non-communicable diseases (NCDs) and mental health disorders are a rapidly increasing health burden in low-and middle-income countries. This study aims to examine the relationships between mental health disorders and cascade of care in managing four common physical NCDs (hypertension, diabetes, dyslipidemia, chronic kidney disease) in China. We utilized two waves of nationally-representative China Health and Retirement Longitudinal Study (CHARLS 2011, 2015) of older adult population aged 45 and above. A series of unadjusted and adjusted mixed-effect logistic regression was applied to evaluate the association between presence of mental health disorder and physical chronic disease awareness, treatment, and control. We found that the odds of dyslipidemia (AOR 1.81, 95% CI 1.36–2.39) and kidney disease awareness (AOR 2.88, 95% CI 2.12–3.92) were higher for individuals with mental chronic conditions, compared to those without mental chronic conditions. The odds of having hypertension treatment was higher for subjects with mental health disorder, compared to those without (AOR 1.32, 95% CI 1.02–1.70). The odds of having physical chronic conditions controlled was not significantly associated with having mental chronic conditions (*P* > 0.05). These results indicated that adults with mental health disorder have a greater likelihood of awareness of having dyslipidemia and kidney disease, and receiving treatment for hypertension. Strategies to address the growing burden of physical-mental NCDs in China should include efforts to improve management of patients with comorbid health condition and improve access to continual high-quality treatment after the first diagnosis.

## Introduction

The prevalence of the co-occurrence of physical non-communicable disease (NCDs) and mental health disorder is increasing rapidly in many low-and middle-income countries (LMIC)^1^. Evidence suggests that the relationship between physical NCDs and mental health disorders is bidirectional^2^. China, the most populous country, has seen rapid demographic and epidemiological transition over the last few decades. By 2050, the projected proportion of the older population, aged 60 years and over, will dramatically increase to 35.1% from 16.2% in 2017^3^. The repaid aging population has led to substantial increase in the prevalence of physical NCDs. For example, a recent study, based on 11 physical NCDs, found that the prevalence of physical multimorbidity was 62% among those aged 45 years and older^4^. The prevalence of mental health disorders has also been increasing in recent decade^5^. Analysis using China Mental Health Survey, based on 32,552 participants in 31 provinces in China, found that the prevalence of any mental health disorders was 9.3% in 2013 among Chinese adults^6^. The rising prevalence of physical and mental chronic conditions is expected to post significant challenges to the health system in China.

The potential impact of mental health disorder on the cascade of care in managing physical NCDs is an emerging area of research interest. The UK National Institute from Health and Care Excellence (NICE) guidelines on multimorbidity have emphasized the challenge of poor management of chronic conditions in adults, which could subsequently be associated with higher treatment burden and poorer health-related quality of life (HRQoL)^7,8^. The literature on the impact of having multimorbidity on the management and control of each of the chronic conditions is scarce, with current studies largely from High-income countries (HICs) that use qualitative methods^9–13^. There is also some quantitative research in the literature that have investigated how having more comorbid NCDs influences management and control of specific conditions, primarily hypertension^14,15^. Furthermore, a limited number of studies, mostly from HICs as well, have focused on the differential impact of concordant versus discordant conditions on the quality of chronic disease management^8,16–20^. While it may be hypothesized that concordant conditions would facilitate the management of a NCD due to the similar pathophysiology and treatment regiments, and discordant NCDs would have opposite effects, existing studies demonstrate inconsistencies on how concordant and discordant NCDs influence the management of chronic diseases^8,17,20^.

To illustrate, a retrospective cohort study in the United States on hypertensive primary care patients examined the impact of concordant versus discordant NCDs of hypertension, on hyperlipidemia management^16^. This study showed that additional discordant conditions were associated with lower odds of hyperlipidemia management compared with no discordant NCDs, and additional concordant conditions were associated with increased odds of hyperlipidemia management^16^. Another study analyzed 15,000 patients from primary care practices in the United States who have multimorbidity and uncontrolled hypertension, and found that patients with more discordant NCDs to hypertension (28 conditions including arthritis and emphysema) were less likely to have their uncontrolled hypertension solved, compared to those with concordant conditions^18^. Our recent paper which also analyzed the World Health Organization study on global aging and adult health data (WHO SAGE) found that having more NCDs was associated with better odds of diagnosis but not better management and control of co-occurring NCDs^21^. Importantly, generating more research from LMICs is crucial as the findings from HICs may not able applicable, due to different health systems and access to healthcare for diagnostic testing and treatment^9,13,17,22^. In addition, impact of a broader range of NCDs needs to be investigated^8,14,15,17,19,20^.

This study examines individuals in China with multimorbidity that consists of both physical and mental health chronic conditions. Specifically, we investigate how having mental health conditions is associated with cascade of care in managing four common physical NCDs (e.g. hypertension, diabetes, dyslipidemia, chronic kidney disease) of the elder adult population in China.

## Methods

### Participants and sample

This study analysed pooled cross-sectional data of the first wave (2011) and third wave (2015) of China Health and Retirement Longitudinal Study (CHARLS). We have registered in CHARLS website and get the permission from Peking University of using this dataset. All participants of CHARLS have signed consent forms before face-to-face interview. Personal information, like ID, address, are removed from the dataset and coded as a series of numbers.

The CHARLS consists of nationally representative participants aged 45 or older from 450 randomly selected communities/villages in 28 provinces of China^23^. It collects information on demographics, family, health status and functioning, health care and insurance, income, work, and housing. Respondents of CHARLS are followed up every two years via face-to-face computer-assisted personal interview (CAPI)^23^. Biomarkers for chronic diseases were used in each wave, while blood tests were conducted every two waves^24^. Additional information about CHARLS are available on http://charls.ccer.edu.cn/.

17,708 subjects participated in the first wave (2011) and 20,967 participated in the third wave (2015). For those participants, 14,574 participated in both the first and third wave (2015). For the purpose of the study, we only include those with biomarker and blood test result (63% of the total observations in the two waves). After removing subjects with missing information on covariates (30% of the sample), the final sample size in this study is 17,141, which include 8189 observations in the first wave and 8952 observations in the third wave.

### Variables

#### Predictor variables

The predictor variable in this study is having a mental health disorder. Having mental health disorder was defined as the existence of any one of those following conditions (1) answer affirmatively to “have you been diagnosed with emotional, nervous, or psychiatric problem by a doctor?” (2) answered affirmatively to “have you been diagnosed with memory-related disease by a doctor?” (3) 10-item Center for Epidemiologic Studies Depression Scale (CES-D-10) higher than 12^25^. This study only included respondents that have answered at least 9 items on CES-D-10, and used the average of the remaining 9 items to impute the missing item.

### Outcome variables

#### Awareness

Having awareness of the chronic condition is defined as being diagnosed by doctors or being self-aware of their chronic conditions. For hypertension, blood pressure was measured three times for each participant and this study only included participants with at least two results on systolic blood pressure and diastolic blood pressure to minimize measurement error. This study revised the WHO definition for hypertension to meet the questionnaire design^26^. Participants with hypertension are defined as the existence of one the following conditions: (1) average systolic blood pressure ≥ 140 mmHg; (2) average diastolic blood pressure ≥ 90 mmHg; (3) self-report hypertension diagnosed by a doctor or self-report aware of hypertension. Hypertension awareness is defined as respondents with hypertension and their hypertension is diagnosed by doctors or they are aware of their hypertension condition. Diabetes is defined as: (1) fasting blood glucose ≥ 126 mg/dl; (2) non-fasting blood glucose ≥ 200 mg/dl; (3) HbA1c concentration ≥ 6.5%; (4) self-report diabetes diagnosed by a doctor^27^. Dyslipidemia is defined as: (1) triglyceride ≥ 200 mg/ml; (2) HDL (high density lipoprotein cholesterol) < 40 mg/dl; (3) LDL (low density lipoprotein cholesterol) ≥ 160 mg/dl; (4) TC (total cholesterol) ≥ 240 mg/dl; (5) self-report dyslipidemia diagnosed by a doctor^28^. Kidney disease is defined as: (1) GFR (Glomerular Filtration Rate) < 90 ml/min/1.72m^2^; (2) with self-report kidney disease diagnosed by a doctor. GFR is calculated by using Levey’s method^29^. Diabetes, dyslipidemia, kidney disease awareness was defined as respondents with diabetes, dyslipidemia, or kidney disease and these conditions are diagnosed by doctors.

#### Treatment

Being treated for a NCD is defined as respondents with self-reported chronic conditions diagnosed by a doctor or being aware of their chronic condition and taking at least one treatment for this chronic condition. For hypertension, respondents answer affirmatively to “taking Chinese traditional medicine” or “taking western modern medicine” to the question “Are you taking any of the following treatments to treat hypertension or its complication?” are considered to be with hypertension treated. For diabetes and kidney disease, respondents choose any one of the following choices “taking Chinese traditional medicine”, “taking western modern medicine” or “other treatments” to question “are you taking any of the following treatment to treat (…) or its complications?” are considered to be with hypertension/kidney disease treatment. For diabetes, respondents choose any one of those choices “taking Chinese traditional medicine” “taking western modern medicine” or “taking insulin injections” to the question “are you taking any of the following treatments to treat or control your diabetes” are considered to be with diabetes treated.

#### Controlled

Being controlled for a NCD is defined as respondents with self-report chronic condition, with self-report treatment and with normal biomarker or blood test result. The clinical criteria for hypertension, dyslipidemia, diabetes, and kidney disease are mentioned in the part of awareness.

#### Covariates

Covariates are age (45–54, 55–64, 65–74, 75 +), sex (male, female), marital status (married, single), education level (primary school or below, middle or high school, college or above), consumption quantile (as a proxy for economic status, is calculated by dividing individual household consumption into 5 equal parts by using 20th, 40th, 60th, and 80th percentiles), residence type (rural, urban, migrates), health insurance status (no insurance, UEMI (Urban Employee Medical Insurance), URMI (Urban Resident Health Insurance), NCMI (New Cooperative Medical Insurance), others), region (eastern, central, western, and north–east), number of outpatient visits, and number of physical chronic conditions (0, 1, 2, 3 ,4+).

### Statistical analysis

Demographic characteristics of participants for year 2011 and 2015 were calculated separately. The demographic characteristics of participants with hypertension, diabetes, dyslipidemia, and or kidney disease were calculated separately for subjects with mental health disorder and without mental health disorder.

We estimated the prevalence of NCD awareness, treatment, and control for subjects with and without mental health disorder separately. Pearson chi-square test was used to test the significance in the difference between subjects with mental health disorder and those without mental health disorder for their awareness, treatment, and control of physical chronic conditions.

A series of unadjusted and adjusted mixed-effects logistics regressions were used to evaluate the effect of mental health disorders on NCD awareness, treatment, and control. People who live in the same community are more likely to have similar living environments and medical conditions, which results in a similarity in chronic disease management. To reduce the impact of unobserved individual characteristics and community characteristics on the estimation of coefficients, this study used fixed effect on the level of individual ID and community IC, while using random effect to estimate the effect of other covariates^30^. First, we conducted unadjusted mixed effect logistics regression with NCD awareness, treatment, or control as the outcome variable and mental health disorder as the predicting variable. Subsequently, we fitted multiple mixed effect logistics regression with NCD awareness, treatment, or control as the outcome variable, mental health disorder as the exposure variable, and adjusted for the covariates mentioned above including frequency of outpatient visits.

All data analysis was performed by using Stata 14.0 and level of significance was set at 0.05.

## Results

The number of observations in this study is 17,141, of which 8189 observations are from year 2011, and 8952 are from year 2015. The demographic characteristics of participants are presented in Table 1 separately for year 2011, year 2015, and overall. Among those observations, 7361 observations with hypertension, 2342 observations with diabetes, 7054 observations with dyslipidemia, and 9265 observations with kidney disease. The demographic characteristics of observations with hypertension, with diabetes, with dyslipidemia, and with kidney disease are presented in Appendix Table 1a,b,c,d.

**Table 1:** Demographic characteristic participants in year 2011, 2015 and overall sample.

	2011 (N = 8189)		2015 (N = 8952)		All (N = 17,141)	
	n	%	n	%	n	%
**Gender**						
Male	3841	46.90	4271	47.71	8112	47.33
Female	4348	53.10	4681	52.29	9029	52.67
**Age**						
45–54	2843	34.72	2564	28.64	5407	31.54
55–64	3218	39.30	3467	38.73	6685	39.00
65–74	1589	19.40	2242	25.04	3831	22.35
75 +	539	6.58	679	7.58	1218	7.11
**Education**						
Middle school or low	5736	70.05	5947	66.43	11,683	68.16
High school/vocational school	2205	26.93	2672	29.85	4877	28.45
College or above	248	3.03	333	3.72	581	3.39
**Marital status**						
Married	7254	88.58	7886	88.09	15,140	88.33
Single	935	11.42	1066	11.91	2001	11.67
**Consumption**						
Q1 (most deprived)	2134	26.06	1880	21.00	4014	23.42
Q2	1997	24.39	1944	21.72	3941	22.99
Q3	1683	20.55	1835	20.50	3518	20.52
Q4	1374	16.78	1757	19.63	3131	18.27
Q5 (most affluent)	1001	12.22	1536	17.16	2537	14.80
**Insurance**						
No	443	5.41	91	1.02	534	3.12
UEMI	632	7.72	1087	12.14	1719	10.03
URMI	305	3.72	488	5.45	793	4.63
NCMI	6534	79.79	7135	79.70	13,669	79.74
Others	275	3.36	151	1.69	426	2.49
**Residence type**						
Rural	5188	63.35	5329	59.53	10,517	61.36
Urban	1202	14.68	1507	16.83	2709	15.80
Migrants	1799	21.97	2116	23.64	3915	22.84
**Region**						
Eastern region	2650	32.36	2657	29.68	5307	30.96
Central region	1471	17.96	1715	19.16	3186	18.59
Western region	3841	46.90	4393	49.07	8234	48.04
North-eastern region	227	2.77	187	2.09	414	2.42

*UEMI* urban employee medical insurance, *URMI* urban resident medical insurance, *NCMI* new cooperative medical insurance.

This table provides the distribution of demographic characteristics of participants in year 2011, 2015, and overall.

The prevalence of NCD awareness, treatment, and control for subjects with and without mental health disorder are presented in Table 2. The prevalence of NCD awareness ranges from 74.47% for people with hypertension and with mental health disorder to 27.71% for people with kidney disease and without mental health disorder. For all of the four NCDs, the prevalence of NCD awareness is significantly higher for people with mental health disorder than people without mental health disorder (*P* < 0.05).

**Table 2:** Prevalence of NCD aware, treated, and controlled for people with and without mental health disorder.

	With mental health disorder			Without mental health disorder			Pearson chi2	*P*-value
	n	N	%	n	N	%		
**Hypertension**								
Aware	1435	1927	74.47	3644	5434	67.06	36.50	< 0.001
Treated	1145	1435	79.79	2762	3644	75.80	9.26	0.002
Controlled	512	1145	44.72	1221	2762	44.25	0.09	0.771
**Diabetes**								
Aware	399	669	59.64	898	1673	53.68	6.88	0.009
Treated	274	399	68.67	633	898	70.49	0.43	0.510
Controlled	123	274	44.89	232	633	36.65	5.45	0.020
**Dyslipidemia**								
Aware	686	1800	38.11	1456	5254	27.71	68.56	< 0.001
Treated	437	686	63.70	782	1456	53.71	18.99	< 0.001
Controlled	215	437	49.20	368	782	47.06	0.51	0.473
**Kidney disease**								
Aware	469	2363	19.85	715	6902	10.36	142.18	< 0.001
Treated	263	469	56.08	348	715	48.67	6.2	0.013
Controlled	124	263	47.15	148	348	42.53	1.29	0.255

n: number of people in this group; N: total number of people in this group. *P* < 0.01, *P* < 0.05, *P* < 0.1. This table compare the significance in the difference of NCD awareness, treatment, and control for people with and without mental health disorders. Chi-square test is used to test the significance in difference.

For hypertension, dyslipidemia, and kidney disease, the prevalence of those physical conditions being treated is higher for people with mental health disorder than without mental health disorder, while this difference is not significant for diabetes (*P*-value = 0.510). For diabetes, the prevalence of physical conditions being controlled is lower for people without mental health disorder than with mental health disorder (*P*-value = 0.020), however, this difference is not significant for the other three physical chronic conditions. Although the prevalence of physical conditions being controlled is lower for people without mental health disorder than people with mental health disorder, the difference is not significant at the 5% level.

Table 3 presents the univariate and multivariate mixed effect logistic regression result. The reference group is people without mental health disorder. Having mental health disorder was associated with increased odds of NCD awareness for dyslipidemia (AOR (Adjusted odds ratio) = 2.88, 95% CI (confidence interval) = 2.21–3.74) and kidney disease (AOR 4.14, 95% CI 2.95–5.81). However, there was not significant increase the odds of hypertension and diabetes awareness (*P* > 0.05). Having mental health disorder was associated with an increased odds of receiving hypertension treatment (AOR 1.32, 95% CI 1.02–1.70), but not diabetes treatment, dyslipidemia treatment, and kidney disease treatment (*P* > 0.05). Having mental health disorder is not associated with increased or decreased odds of being controlled for all four NCDs (*P* > 0.05). All regression results are listed in the appendix Table 1.

**Table 3:** Effect of mental health disorder on NCD awareness, treatment, and control.

	Without mental health disorder	95% CI	With mental health disorder	95% CI
**Hypertension**				
Aware (N = 9542)				
Unadjusted-OR	1 (Ref)	–	1.94***	(1.53–2.46)
Adjusted-OR	1 (Ref)	–	1.18	(0.92–1.52)
Treated (N = 6624)				
Unadjusted-OR	1 (Ref)	–	1.57***	(1.23–2.01)
Adjusted-OR	1 (Ref)	–	1.32**	(1.02–1.70)
Controlled (N = 4203)				
Unadjusted-OR	1 (Ref)	–	1.03	(0.85–1.25)
Adjusted-OR	1 (Ref)	–	1.05	(0.86–1.28)
**Diabetes**				
Aware (N = 2702)				
Unadjusted-OR	1 (Ref)	–	2.00***	(1.36–2.94)
Adjusted-OR	1 (Ref)	–	1.18	(0.76–1.83)
Treated (N = 1675)				
Unadjusted-OR	1 (Ref)	–	0.97	(0.60–1.45)
Adjusted-OR	1 (Ref)	–	0.92	(0.55–1.54)
Controlled (N = 886)				
Unadjusted-OR	1 (Ref)	–	1.69**	(1.03–2.76)
Adjusted-OR	1 (Ref)	–	1.23	(0.75–2.00)
**Dyslipidemia**				
Aware (N = 7519)				
Unadjusted-OR	1 (Ref)	–	2.88***	(2.21–3.74)
Adjusted-OR	1 (Ref)	–	1.81***	(1.36–2.39)
Treated (N = 2756)				
Unadjusted-OR	1 (Ref)	–	1.74***	(1.33–2.26)
Adjusted-OR	1 (Ref)	–	1.29*	(0.99–1.68)
Controlled (N = 1168)				
Unadjusted-OR	1 (Ref)	–	1.07	(0.72–1.60)
Adjusted-OR	1 (Ref)	–	0.93	(0.59–1.45)
**Kidney disease**				
Aware (N = 9218)				
Unadjusted-OR	1 (Ref)	–	4.14***	(2.95–5.81)
Adjusted-OR	1 (Ref)	–	2.88***	(2.12–3.92)
Treated (N = 1528)				
Unadjusted-OR	1 (Ref)	–	1.50**	(1.09–2.05)
Adjusted-OR	1 (Ref)	–	1.35*	(0.97–1.88)
Controlled (N = 575)				
Unadjusted-OR	1 (Ref)	–	1.21	(0.67–2.18)
Adjusted-OR	1 (Ref)	–	1.04	(0.56–1.91)

*OR* odds ratio *CI* confidence interval. ***P < 0.01, **P < 0.05, *P < 0.1. This table provides the results from the unadjusted and adjusted mixed-effect logistics regression. For awareness, people with chronic physical condition and without mental health disorder was the reference group. For treated, people with self-report physical chronic condition and without mental health disorder was the reference group. For controlled, people with self-report physical condition, and with self-report treatment for this chronic condition, but without mental health disorder was the reference group. Adjusted mixed-effect logistic regressions adjusted variables, such as age, gender, education, marital status, consumption level, insurance, residency, region, and number of outpatient visits in the last month.

## Discussion

### Principle findings

We presented the first study that investigate the associations between having mental health disorder and cascade of care in managing four common physical NCDs in China among the older adult population. Our study revealed that having mental health disorder was associated with increased odds of being aware of having dyslipidemia, and kidney disease, after adjusting for covariates including frequency of outpatient visit. Additionally, having mental health disorder was associated with increased odds of receiving treatment of hypertension, but not for diabetes, dyslipidemia, and kidney disease. However, having mental health disorder was not associated with increased or decreased odds of being controlled for hypertension, diabetes, dyslipidemia and kidney disease.

### Comparison with literature

The finding on the positive effect of having mental health conditions on better diagnosis of previously undiagnosed dyslipidemia and kidney disease is consistent with the small number of existing articles. Subjects with more comorbidities likely resulted in having more frequent visits to and interactions with multiple health providers^8,20,31–33^, such as dyslipidemia, and kidney disease in this particular study. Additionally, healthcare providers are likely to spend more time on consultation and suggest more examinations for patients with multiple chronic conditions. Third, healthcare providers often prescribe thyroid function and blook lipid tests for patients with mental health disorders, because they all believe thyroid function is strongly associated with emotional problems and patients with thyroid imbalance are more likely to develop lipid problems^34^. Fourth, for patients with long-term treatment of psychotropic drugs, healthcare providers often suggest them to do liver and kidney function tests regularly, because long-term use of medication may cause liver or kidney function damage^35^. An increased frequency of healthcare visits, having more comorbidities, and more interactions with healthcare providers were likely associated with a greater tendency for patients to self-report kidney pain and test blood liquid^36,37^.

Studies that have examined the relationship between comorbid health condition and NCD treatment, have shown conflicting results which may reflect complexity of the issue^8,20,33,38–40^. It is worth noting that our study considered only whether subjects were taking treatment or not, and did not if treatment was adequate, in terms of adherence to medication^8,20,41^. While our study showed that subjects with mental health disorder have higher odds of taking treatment, but in reality, with more co-occurring physical conditions, the odds of treatment adherence and having adequate treatment would decline^8,20^.

The finding on having mental health conditions not associated with increased or decreased odds of being controlled for hypertension, diabetes, dyslipidemia and kidney disease is not consistent with the little amount of existing literature. The difficulty in controlling NCDs tend to be exacerbated with having more co-occurring physical chronic conditions^12,14,18^.

There has been debate in the recent literature on how co-occurring conditions influence the management and control of NCDs^8,20^. Magnan et al. (2014) analyzed electronic health data records of 24,430 adults aged 18 to 75 years from the United States, and revealed that even though having more concordant NCDs were correlated with a higher likelihood of achieving diabetes control goals, this relationship was not present for the outcome on achieving blood pressure control^19^. Ricci-Cabello et al. (2015) investigated the prevalence of concordant and discordant NCDs of diabetes, and their impact on diabetes care in England^8,17^. The study revealed that only 2 of 8 discordant NCDs to diabetes were correlated with worse quality of diabetes care, and only 4 of 7 concordant NCDs with diabetes were correlated with better quality of diabetes care^17^.

Hence, this study along with our previous work and other papers, provide further evidence on the complexity of how co-occurring mental health conditions impact the management and control of NCDs, and the hypothesis that concordant comorbidities with mental illness facilitate the management of NCDs and discordant comorbidities with mental illness impede the management of NCDs may be over-simplified^8,20^.

### Strengths and limitations

This is the first study that used a large population of adults from China with multimorbidity, to investigate the association between having mental health conditions with the odds of being undiagnosed, untreated and uncontrolled for co-occurring physical chronic condition.

However, several limitations need to be considered when drawing conclusion from this study. First, Self-reported diagnosis and treatment of chronic conditions may be under-reported due to recall bias^42–44^. Stigma could be another reason for under-reporting of depression in MICs^45,46^. Additionally, this survey only asked if subjects were taking treatment (medicines, lifestyle changes), but did not measure the adherence to treatment (i.e. dosage, frequency, duration, etc.)^20,41^. Biomarkers used to assess whether chronic conditions were controlled may not be sufficiently comprehensive. Supplemental assessment criteria may have been needed for better accuracy. Third, results from this study may not apply to those excluded from our study due to incomplete data of biomarkers or blood test results.

Future studies could expand on this study by examining more NCDs, especially conditions with high prevalence and morbidity^8,20,47^. The study’s cross-sectional design means that causality could not be determined, and studies that use cohort study designs could examine how mental health conditions lead to worse treatment and control of physical chronic diseases in subjects that are followed-up over a decade^8,20,38^.

### Clinical, policy, and research implications

Clinical guidelines must be updated to include improved screening and treatment of physical chronic conditions that may occur in patients with mental health conditions^8,20^. Regarding poorer treatment and control of mental health conditions and physical chronic diseases associated with having more NCDs, clinical guidelines could incorporate more intentional monitoring of patients’ adherence to medication and treatments by clinicians^8,20^. Also proposed in our previous work, current clinical guidelines are based on evidence from controlled trials for treating single NCDs^8,20,48–50^, and treatments may no longer be effective and even have adverse effects when applying single-disease guidelines to patients with multimorbidity^8,20,51^. Hence, clinical guidelines should be tailored towards an approach to multimorbidity of co-occurring mental illness and physical chronic conditions, whereby clinicians review the effectiveness and risks of combining the medications and different treatments for mental illness and physical chronic diseases^7,8,20,48^.

Healthcare systems in LMICs like China may need to implement policies that improve access to care from the primary care system for continual treatment after first diagnosis^52–55^. Policies that prioritize NCD combinations that include mental health conditions that are more prevalent or associated with poorer management and control need be considered, such as reducing costs of medicines and clinic visits^56,57^. It is worth noting that health-care delivery in China is hospital-centered and fragmented, with little coordination among health-care providers across different tiers of the system^57,58^. Strong primary health care, underpinned by multidisciplinary teams lead by general practitioner, is also crucial for the improved prevention and treatment of patients with multiple NCDs. Health care delivery need to shift away from the current vertical approach of treating single-disease models to one that emphasize on horizontal integration that aims to provide more effectively management for patients with multiple NCDs, including co-existing physical and mental NCDs. Overall, our study provides evidence on the impact of comorbid mental health condition on the management of physical NCDs in China. Further research is required to better understand the epidemiology of co-existing mental-physical NCDs and associated impacts on management of the conditions and associated costs and health outcomes in LMIC settings.

## Supplementary Information

Supplementary information.

## References

[1] Mendenhall E, Kohrt BA, Norris SA, Ndetei D, Prabhakaran D (2017). Non-communicable disease syndemics: poverty, depression, and diabetes among low-income populations. Lancet.

[2] Momen NC (2020). Association between mental disorders and subsequent medical conditions. N. Engl. J. Med..

[3] Day, J. C. *Population projections of the United States, by age, sex, race, and Hispanic origin: 1992 to 2050*. (US Department of Commerce, Economics and Statistics Administration, Bureau …, 1992).

[4] Zhao Y (2020). Physical multimorbidity, health service use, and catastrophic health expenditure by socioeconomic groups in China: an analysis of population-based panel data. Lancet Glob. Health.

[5] Lu S, Oldenburg B, Li W, He Y, Reavley N (2019). Population-based surveys and interventions for mental health literacy in China during 1997–2018: a scoping review. BMC Psychiatry.

[6] Huang Y (2019). Prevalence of mental disorders in China: a cross-sectional epidemiological study. Lancet Psychiatry.

[7] Eulenburg C (2016). Propensity scoring after multiple imputation in a retrospective study on adjuvant radiation therapy in lymph-node positive vulvar cancer. PLoS ONE.

[8] Sum, G. Patient centric perspectives on the public health burden of multimorbidity: Out of pockey expenditure, work productivity, healthcare utilisation, quality of life, and disease management and control. *SSHSPH NUS* (2020).

[9] Noel PH, Frueh BC, Larme AC, Pugh JA (2005). Collaborative care needs and preferences of primary care patients with multimorbidity. Health Expect.

[10] Noel PH (2007). The challenges of multimorbidity from the patient perspective. J Gen Intern Med.

[11] Bayliss EA, Steiner JF, Fernald DH, Crane LA, Main DS (2003). Descriptions of barriers to self-care by persons with comorbid chronic diseases. Ann. Fam. Med..

[12] Piette JD, Kerr EA (2006). The impact of comorbid chronic conditions on diabetes care. Diabetes Care.

[13] Bair MJ (2009). Barriers and facilitators to chronic pain self-management: a qualitative study of primary care patients with comorbid musculoskeletal pain and depression. Pain Med.

[14] Wong MC (2014). Factors associated with multimorbidity and its link with poor blood pressure control among 223,286 hypertensive patients. Int. J. Cardiol..

[15] Li YT (2016). Medication adherence and blood pressure control among hypertensive patients with coexisting long-term conditions in primary care settings: a cross-sectional analysis. Medicine.

[16] Lagu T (2008). The impact of concordant and discordant conditions on the quality of care for hyperlipidemia. J. Gen. Int. Med..

[17] Ricci-Cabello I (2015). Impact of the prevalence of concordant and discordant conditions on the quality of diabetes care in family practices in England. Ann. Fam. Med..

[18] Turner BJ, Hollenbeak CS, Weiner M, Ten Have T, Tang SS (2008). Effect of unrelated comorbid conditions on hypertension management. Ann. Int. Med..

[19] Magnan EM (2015). The impact of a patient's concordant and discordant chronic conditions on diabetes care quality measures. J. Diabetes Comp..

[20] Sum G (2020). Patients with more comorbidities have better detection of chronic conditions, but poorer management and control: findings from six middle-income countries. BMC Public Health.

[21] Sum G (2020). Patients with more comorbidities have better detection of chronic conditions, but poorer management and control: findings from six middle-income countries. BMC Public Health.

[22] Palladino R, Tayu Lee J, Ashworth M, Triassi M, Millett C (2016). Associations between multimorbidity, healthcare utilisation and health status: evidence from 16 European countries. Age Age..

[23] Zhao, Y. *et al.* China health and retirement longitudinal study–2011–2012 national baseline users’ guide. *Beijing: National School of Development, Peking University*, 1–56 (2013).

[24] Zhao Y, Hu Y, Smith JP, Strauss J, Yang G (2014). Cohort profile: the China health and retirement longitudinal study (CHARLS). Int. J. Epidemiol..

[25] Luo H (2018). Obesity and the onset of depressive symptoms among middle-aged and older adults in China: evidence from the CHARLS. BMC Public Health.

[26] Alwan, A. *Global status report on noncommunicable diseases 2010*. (World Health Organization, 2011).

[27] Zhao Y (2016). Prevalence, diagnosis, and management of diabetes mellitus among older Chinese: results from the China Health and Retirement Longitudinal Study. Int. J. Public Health.

[28] Adults I (2016). Chinese guideline for the management of dyslipidemia in adults. Zhonghua Xin Xue Guan Bing Za Zhi.

[29] Levey AS (2002). K/DOQI clinical practice guidelines for chronic kidney disease: evaluation, classification, and stratification. Am. J. Kidney Dis..

[30] Liu Y, Dijst M, Faber J, Geertman S, Cui C (2017). Healthy urban living: residential environment and health of older adults in Shanghai. Health Place.

[31] Zhang Y, Moran AE (2017). Trends in the prevalence, awareness, treatment, and control of hypertension among young adults in the United States, 1999 to 2014. Hypertension.

[32] Mirza AA, Elmorsy SA (2016). Diagnosis and Control of Hypertension as Indicators of the Level of Awareness Among Relatives of Medical Students in Saudi Arabia. High Blood Pressure Cardiovasc. Prevent. Off. J. Italian Soc. Hypertension.

[33] Pasina L (2014). Medication non-adherence among elderly patients newly discharged and receiving polypharmacy. Drugs Aging.

[34] Kafle B, Khadka B, Tiwari ML (2020). Prevalence of thyroid dysfunction among depression patients in a tertiary care centre. JNMA J. Nepal Med. Assoc..

[35] Lala, V., Goyal, A., Bansal, P. & Minter, D. Liver function tests. *StatPearls* (2020). 29494096

[36] McConaghy JR, Oza R (2013). Outpatient diagnosis of acute chest pain in adults. Am. Fam. Phys..

[37] Shima A (2016). Relationship between outpatient visit frequency and hypertension control: a 9-year occupational cohort study. Hypertens. Res..

[38] Chow CK (2013). Prevalence, awareness, treatment, and control of hypertension in rural and urban communities in high-, middle-, and low-income countries. JAMA.

[39] Gellad WF, Grenard JL, Marcum ZA (2011). A Systematic Review of Barriers to Medication Adherence in the Elderly: Looking Beyond Cost and Regimen Complexity. Am. J. Geriatr. Pharmacother..

[40] Sum G (2018). Multimorbidity and out-of-pocket expenditure on medicines: a systematic review. BMJ Glob Health.

[41] Brown MT, Bussell JK (2011). Medication adherence: WHO cares?. Mayo Clin. Proc..

[42] Chatterji, S. & Kowal, P. (Inter-university Consortium for Political and Social Research [distributor], 2013).

[43] Kowal P (2012). Data resource profile: the World Health Organization Study on global AGEing and adult health (SAGE). Int. J. Epidemiol..

[44] Vellakkal S (2015). Are estimates of socioeconomic inequalities in chronic disease artefactually narrowed by self-reported measures of prevalence in low-income and middle-income countries? Findings from the WHO-SAGE survey. J. Epidemiol. Commun. Health.

[45] Busby Grant J, Bruce CP, Batterham PJ (2016). Predictors of personal, perceived and self-stigma towards anxiety and depression. Epidemiol. Psychiatr. Sci..

[46] Jones AR, Cook TM, Wang J (2011). Rural-urban differences in stigma against depression and agreement with health professionals about treatment. J. Affect. Disord..

[47] Barnett K (2012). Epidemiology of multimorbidity and implications for health care, research, and medical education: a cross-sectional study. Lancet.

[48] Kovacevic SV (2017). Evaluation of drug-related problems in older polypharmacy primary care patients. J. Eval. Clin. Pract..

[49] Feng XQ, Zhu LL, Zhou Q (2017). Opioid analgesics-related pharmacokinetic drug interactions: from the perspectives of evidence based on randomized controlled trials and clinical risk management. J. Pain Res..

[50] Vrdoljak D, Borovac JA (2015). Medication in the elderly - considerations and therapy prescription guidelines. Acta Med. Acad..

[51] Stewart D (2017). Guidance to manage inappropriate polypharmacy in older people: systematic review and future developments. Expert Opin. Drug Saf..

[52] Frieden TR (2010). A framework for public health action: the health impact pyramid. Am. J. Public Health.

[53] Waibel S, Vargas I, Coderch J, Vázquez M-L (2018). Relational continuity with primary and secondary care doctors: a qualitative study of perceptions of users of the Catalan national health system. BMC Health Serv. Res..

[54] Wright M, Mainous AG (2018). Can continuity of care in primary care be sustained in the modern health system?. Aust. J. Gen. Pract..

[55] Guthrie B (2002). Continuity in UK general practice: a multilevel model of patient, doctor and practice factors associated with patients seeing their usual doctor. Fam. Pract..

[56] Chen H, Chen Y, Cui B (2018). The association of multimorbidity with healthcare expenditure among the elderly patients in Beijing China. Arch. Gerontol. Geriatr..

[57] Sum G (2018). Multimorbidity and out-of-pocket expenditure on medicines: a systematic review. BMJ global health.

[58] Wang X (2018). People-centred integrated care in urban China. Bull. World Health Organ..

