# Supplementary material for: Associations between mental health disorder and management of physical chronic conditions in China: a pooled cross-sectional analysis
Source: Sci Rep. 2021 Mar 11;11:5731. doi: 10.1038/s41598-021-85126-4 (PMC7952541; doi:10.1038/s41598-021-85126-4)
Supplement: Supplementary file 1 — Supplementary information. [file 41598_2021_85126_MOESM1_ESM.pdf]

Associations between mental health disorder and management of physical chronic conditions in China: a pooled cross-sectional analysis

Zheng Zhang[1*], Grace Sum[2], Vicky Mengqi Qin[2], Yang Zhao[3,4],Tilahun Nigatu Haregu[4,5],Brian Oldenburg[4,5],John Tayu Lee[5,6]

[1] School of Population and Global Health, University of Melbourne, VIC, Australia
[2] Saw Swee Hock School of Public Health, National University of Singapore, Singapore
[3] The George Institute for Global Health at Peking University Health Science Centre, Beijing, China
[4] WHO collaborating Centre on Implementation Research for Prevention and Control of Noncommunicable Disease, Melbourne, VIC, Australia
[5] The Nossal Institute for Global Health, The University of Melbourne, Melbourne, VIC, Australia
[6] Department of Primary Care and Public Health, School of Public Health, Imperial College, London,

*Correspondence to: zheng zhang zhengz7@student.unimelb.edu.au

Grace Sum gracesum@u.nus.edu
Vicky Mengqi Qin mq.qin@u.nus.edu
Yang Zhao xiyunjushi@163.com
Tilahun Nigatu Haregu haregu.t@unimelb.edu.au
Brain Oldenburg brian.oldenburg@unimelb.edu.au
John Tayu Lee johntayulee@unimelb.edu.au

Appendix graph 1: flow chart

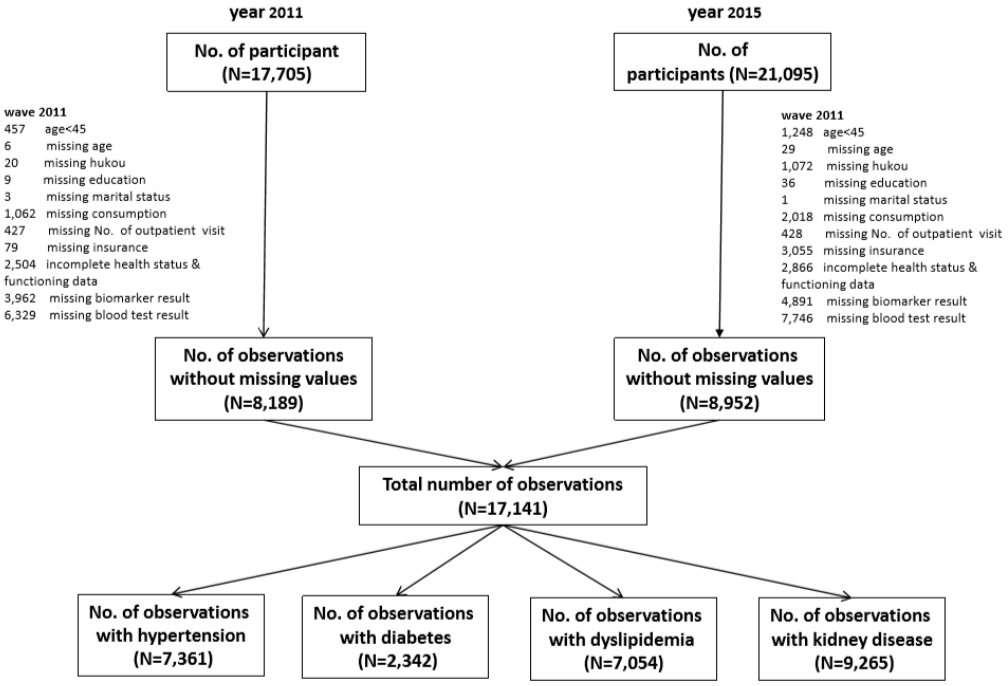

Table 1a. Demographic characteristic of people with hypertension, self-report hypertension, and self-report hypertension treatment

	With hypertension (N=7,361)		With self-report hypertension (N=5,079)		With self-report hypertension treatment (N=3,907)	
	n	%	n	%	n	%
**Gender**						
**Male**	3,487	47.37	2,316	45.60	1,728	44.23
**Female**	3,874	52.63	2,763	54.40	2,179	55.77
**Age**						
**45-54**	1,653	22.46	1,083	21.32	765	19.58
**55-64**	2,896	39.34	2,066	40.68	1,600	40.95
**65-74**	2,075	28.19	1,458	28.71	1,164	29.79
**75+**	737	10.01	472	9.29	378	9.67
**Education**						
**Middle school or low**	5,223	70.96	3,556	70.01	2,747	70.31
**High school/vocational school**	1,867	25.36	1,320	25.99	995	25.47
**College or above**	271	3.68	203	4.00	165	4.22
**Marital Status**						
**Married**	6,290	85.45	4,376	86.16	3,365	86.13
**Single**	1,071	14.55	703	13.84	542	13.87
**Consumption level**						
**Q1 (most deprived)**	1,895	25.74	1,206	23.74	936	23.96
**Q2**	1,691	22.97	1,140	22.45	864	22.11
**Q3**	1,479	20.09	1,057	20.81	820	20.99
**Q4**	1,280	17.39	909	17.90	689	17.64
**Q5 (most affluent)**	1,016	13.80	767	15.10	598	15.31
**Insurance**						
**No**	222	3.02	134	2.64	95	2.43
**UEMI**	838	11.38	666	13.11	529	13.54
**URMI**	376	5.11	283	5.57	221	5.66
**NCMI**	5,731	77.86	3,860	76.00	2,953	75.58
**Others**	194	2.64	136	2.68	109	2.79
**Residency**						
**Rural**	4,317	58.65	2,922	57.53	2,195	56.18
**Urban**	1,304	17.71	1,017	20.02	820	20.99
**Migration**	1,740	23.64	1,140	22.45	892	22.83
**Region**						
**Eastern region**	2,349	31.91	1,647	32.43	1,302	33.32
**Central region**	1,417	19.25	980	19.30	748	19.15
**Western region**	3,407	46.28	2,308	45.44	1,750	44.79
**North-eastern region**	188	2.55	144	2.84	107	2.74

Note: UEMI=Urban Employee Medical Insurance; URMI=Urban Resident Medical Insurance; NCMI= New Cooperative Medical Insurance.

Table 1b. Demographic characteristic of people with diabetes, with self-report diabetes, and self-report diabetes treatment

	With diabetes (N=2,342)		With self-report diabetes (N=1,297)		With self-report diabetes treatment (N=907)	
	n	%	n	%	n	%
**Gender**						
**Male**	1,057	45.13	536	41.33	385	42.45
**Female**	1,285	54.87	761	58.67	522	57.55
**Age**						
**45-54**	593	25.32	291	22.44	194	21.39
**55-64**	974	41.59	550	42.41	396	43.66
**65-74**	597	25.49	359	27.68	249	27.45
**75+**	178	7.60	97	7.48	68	7.50
**Education**						
**Middle school or low**	1,566	66.87	844	65.07	589	64.94
**High school/vocational school**	647	27.63	365	28.14	252	27.78
**College or above**	129	5.51	88	6.78	66	7.28
**Marital Status**						
**Married**	2,075	88.60	1,153	88.90	808	89.08
**Single**	267	11.40	144	11.10	99	10.92
**Consumption level**						
**Q1 (most deprived)**	515	21.99	244	18.81	166	18.30
**Q2**	526	22.46	276	21.28	190	20.95
**Q3**	482	20.58	266	20.51	179	19.74
**Q4**	437	18.66	272	20.97	208	22.93
**Q5 (most affluent)**	382	16.31	239	18.43	164	18.08
**Insurance**						
**No**	81	3.46	39	3.01	30	3.31
**UEMI**	328	14.01	231	17.81	170	18.74
**URMI**	143	6.11	92	7.09	64	7.06
**NCMI**	1,717	73.31	892	68.77	607	66.92
**Others**	73	3.12	43	3.32	36	3.97
**Residency**						
**Rural**	1,261	53.84	633	48.80	424	46.75
**Urban**	537	22.93	363	27.99	268	29.55
**Migration**	544	23.23	301	23.21	215	23.70
**Region**						
**Eastern region**	769	32.84	448	34.54	310	34.18

**Central region**	378	16.14	197	15.19	142	15.66
**Western region**	1,136	48.51	609	46.95	423	46.64
**North-eastern region**	59	2.52	43	3.32	32	3.53

Note: UEMI=Urban Employee Medical Insurance; URMI=Urban Resident Medical Insurance; NCMI= New Cooperative Medical Insurance.

Table 1c. Demographic characteristic of people with dyslipidemia, with self-report dyslipidemia, and self-report dyslipidemia treatment

	With dyslipidemia (N=7,054)		With self-report dyslipidemia (N=2,142)		With self-report dyslipidemia treatment (N=1,219)	
	n	%	n	%	n	%
**Gender**						
**Male**	3,296	46.73	963	44.96	542	44.46
**Female**	3,758	53.27	1,179	55.04	677	55.54
**Age**						
**45-54**	2,211	31.34	564	26.33	298	24.45
**55-64**	2,883	40.87	924	43.14	556	45.61
**65-74**	1,521	21.56	538	25.12	304	24.94
**75+**	439	6.22	116	5.42	61	5.00
**Education**						
**Middle school or low**	4,608	65.32	1,272	59.38	775	63.58
**High school/vocational school**	2,128	30.17	710	33.15	365	29.94
**College or above**	318	4.51	160	7.47	79	6.48
**Marital Status**						
**Married**	6,270	88.89	1,913	89.31	1,087	89.17
**Single**	784	11.11	229	10.69	132	10.83
**Consumption level**						
**Q1 (most deprived)**	1,505	21.34	388	18.11	226	18.54
**Q2**	1,582	22.43	456	21.29	271	22.23
**Q3**	1,432	20.30	419	19.56	240	19.69
**Q4**	1,356	19.22	451	21.06	250	20.51
**Q5 (most affluent)**	1,179	16.71	428	19.98	232	19.03
**Insurance**						
**No**	214	3.03	40	1.87	24	1.97
**UEMI**	902	12.79	429	20.03	209	17.15
**URMI**	380	5.39	140	6.54	69	5.66
**NCMI**	5,352	75.87	1,466	68.44	881	72.27
**Others**	206	2.92	67	3.13	36	2.95
**Residency**						
**Rural**	3,928	55.68	1,053	49.16	627	51.44
**Urban**	1,412	20.02	599	27.96	298	24.45
**Migration**	1,714	24.30	490	22.88	294	24.12

Region						
Eastern region	2,207	31.29	710	33.15	402	32.98
Central region	1,412	20.02	388	18.11	232	19.03
Western region	3,249	46.06	974	45.47	548	44.95
North-eastern region	186	2.64	70	3.27	37	3.04

Note: UEMI=Urban Employee Medical Insurance; URMI=Urban Resident Medical Insurance; NCMI= New Cooperative Medical Insurance.

Table 1d. Demographic characteristic of people with kidney disease, with self-report kidney disease, and with self-report kidney disease treatment

	With kidney disease (N=9,265)		With self-report kidney disease (N=1,184)		With self-report kidney disease treatment (N=611)	
	n	%	n	%	n	%
**Gender**						
**Male**	4,277	46.16	634	53.55	333	54.50
**Female**	4,988	53.84	550	46.45	278	45.50
**Age**						
**45-54**	2,295	24.77	338	28.55	177	28.97
**55-64**	3,600	38.86	481	40.63	251	41.08
**65-74**	2,458	26.53	284	23.99	142	23.24
**75+**	912	9.84	81	6.84	41	6.71
**Education**						
**Middle school or low**	6,476	69.90	807	68.16	414	67.76
**High school/vocational school**	2,439	26.32	333	28.13	175	28.64
**College or above**	350	3.78	44	3.72	22	3.60
**Marital Status**						
**Married**	7.989	86.23	1,055	89.10	536	87.73
**Single**	1,276	13.77	129	10.90	75	12.27
**Consumption level**						
**Q1 (most deprived)**	2,152	23.23	232	19.59	113	18.49
**Q2**	2,051	22.14	269	22.72	143	23.40
**Q3**	1,903	20.54	250	21.11	125	20.46
**Q4**	1,734	18.72	214	18.07	107	17.51
**Q5 (most affluent)**	1,425	15.38	219	18.50	123	20.13
**Insurance**						
**No**	271	2.92	23	1.94	10	1.64
**UEMI**	1,118	12.07	138	11.66	60	9.82
**URMI**	460	4.96	80	6.76	45	7.36
**NCMI**	7,156	77.24	909	76.77	477	78.07
**Others**	260	2.81	34	2.87	19	3.11
**Residency**						

**Rural**	5,431	58.62	700	59.12	357	58.43
**Urban**	1,692	18.26	212	17.91	101	16.53
**Migration**	2,142	23.12	272	22.97	153	25.04
**Region**						
**Eastern region**	2,748	29.66	331	27.96	142	23.24
**Central region**	1.697	18.32	292	24.66	181	29.62
**Western region**	4,577	49.40	518	43.75	267	43.70
**North-eastern region**	243	2.62	43	3.63	21	3.44

Note: UEMI=Urban Employee Medical Insurance; URMI=Urban Resident Medical Insurance; NCMI= New Cooperative Medical Insurance.

Table 2a. univariate regression result of hypertension awareness

Hypertension awareness	Odds Ratio	Std.Err	Z	P>|Z|	95% conf. Interval	
**Mental health disorder**						
**yes**	1.94	0.24	5.45	0.000	1.53	2.46
**_cons**	4.10	0.41	14.00	0.000	3.36	4.99

Note: Std.Err= standard error; conf.Interval= confidence interval.

Table 2b. multivariate regression result of hypertension awareness

Hypertension awareness	Odds Ratio	Std.Err	Z	P>|Z|	95% conf. Interval	
**Mental health disorder**						
**yes**	1.31	0.19	1.90	0.057	0.99	1.74
**Year**						
**2015**	3.63	0.46	10.25	0.000	2.84	4.64
**No. of physical comorbidities**						
**One**	3.84	0.60	8.59	0.000	2.82	5.21
**Two**	9.52	1.90	11.28	0.000	6.43	14.08
**Three**	21.32	5.63	11.59	0.000	12.71	35.77
**Four or more**	42.20	13.48	11.71	0.000	22.56	78.93
**Gender**						
**Female**	1.81	0.25	4.36	0.000	1.39	2.37
**Education level**						
**Middle or high school**	1.29	0.22	1.48	0.138	0.92	1.79
**College or above**	1.03	0.40	0.09	0.932	0.48	2.21
**Marital status**						
**Not married**	0.73	0.14	-1.65	0.099	0.51	1.06
**Age**						
**55-64**	1.72	0.28	3.29	0.001	1.24	2.37
**65-74**	1.37	0.26	1.70	0.088	0.95	1.98
**75+**	1.01	0.25	0.02	0.981	0.62	1.64
**Consumption level**						

**Q2**	1.13	0.19	0.75	0.451	0.82	1.57
**Q3**	1.54	0.28	2.43	0.015	1.09	2.19
**Q4**	1.27	0.24	1.24	0.216	0.87	1.85
**Q5 (most affluent)**	1.86	0.40	2.85	0.004	1.21	2.84
**Insurance**						
**UEMI**	1.87	0.78	1.51	0.131	0.83	4.24
**URMI**	1.15	0.53	0.30	0.762	0.47	2.82
**NCMI**	1.12	0.38	0.33	0.743	0.57	2.19
**Others**	1.54	0.78	0.86	0.391	0.57	4.15
**Residency**						
**Urban**	1.95	0.58	2.27	0.023	1.10	3.48
**Migrate**	0.83	0.16	-0.98	0.326	0.57	1.20
**No. of outpatient visit**	1.24	0.07	3.78	0.000	1.11	1.38
**Region**						
**Central**	0.89	0.22	-0.50	0.618	0.55	1.43
**Western**	0.71	0.14	-1.77	0.077	0.49	1.04
**North-east**	1.34	0.73	0.53	0.594	0.46	3.88
**_cons**	0.24	0.10	-3.41	0.001	0.10	0.54

Note: UEMI=Urban Employee Medical Insurance; URMI=Urban Resident Medical Insurance; NCMI= New Cooperative Medical Insurance; Std.Err= standard error; conf.Interval= confidence interval.

Table 2c. univariate regression result of hypertension treated

Hypertension treated	Odds Ratio	Std.Err	Z	P>|Z|	95% conf. Interval	
**Mental health disorder**						
**yes**	1.57	0.20	3.59	0.000	1.23	2.01
**_cons**	6.34	0.82	14.33	0.000	4.92	8.15

Note: Std.Err= standard error; conf.Interval= confidence interval.

Table 2d. multivariate regression result of hypertension treated

Hypertension treated	Odds Ratio	Std.Err	Z	P>|Z|	95% conf. Interval	
**Mental health disorder**						
**Yes**	1.32	0.17	2.13	0.033	1.02	1.71
**Year**						
**2015**	1.01	0.11	0.10	0.920	0.82	1.24
**No. of physical comorbidities**						
**One**	1.23	0.18	1.41	0.158	0.92	1.64
**Two**	1.62	0.27	2.92	0.004	1.17	2.24
**Three**	1.74	0.34	2.88	0.004	1.19	2.55
**Four and more**	2.89	0.64	4.82	0.000	1.88	4.46
**Gender**						
**Female**	1.54	0.19	3.59	0.000	1.22	1.95

**Education level**						
**Middle or high school**	0.99	0.14	-0.06	0.951	0.74	1.32
**College or above**	1.55	0.52	1.29	0.197	0.80	2.99
**Marital status**						
**Not married**	0.82	0.14	-1.14	0.255	0.58	1.15
**Age**						
**55-64**	1.87	0.28	4.19	0.000	1.40	2.51
**65-74**	2.48	0.43	5.18	0.000	1.76	3.49
**75+**	2.86	0.70	4.27	0.000	1.76	4.63
**Consumption level**						
**Q2**	0.78	0.12	-1.53	0.125	0.57	1.07
**Q3**	0.94	0.16	-0.35	0.729	0.68	1.31
**Q4**	0.82	0.15	-1.12	0.263	0.58	1.16
**Q5 (most affluent)**	1.01	0.19	0.03	0.979	0.69	1.47
**Insurance**						
**UEMI**	1.32	0.51	0.73	0.464	0.62	2.81
**URMI**	1.22	0.50	0.49	0.627	0.55	2.73
**NCMI**	1.95	0.66	1.98	0.048	1.01	3.78
**Others**	1.78	0.85	1.20	0.229	0.70	4.56
**Residency**						
**Urban**	2.27	0.60	3.10	0.002	1.35	3.80
**Migrate**	1.64	0.29	2.78	0.005	1.16	2.33
**No. of outpatient**	1.04	0.04	1.01	0.310	0.97	1.12
**Region**						
**Central**	0.89	0.19	-0.56	0.573	0.58	1.35
**Western**	0.77	0.13	-1.51	0.130	0.55	1.08
**North-east**	0.50	0.23	-1.52	0.128	0.20	1.22
**_cons**	1.11	0.45	0.26	0.793	0.50	2.45

Note: UEMI=Urban Employee Medical Insurance; URMI=Urban Resident Medical Insurance; NCMI= New Cooperative Medical Insurance; Std.Err= standard error; conf.Interval= confidence interval.

Table 2e. univariate regression result of hypertension controlled

Hypertension controlled	Odds Ratio	Std.Err	Z	P>|Z|	95% conf. Interval	
**Mental health disorder**						
**yes**	1.03	0.10	0.32	0.750	0.85	1.25
**_cons**	0.74	0.05	-4.90	0.000	0.65	0.83

Note: Std.Err= standard error; conf.Interval= confidence interval.

Table 2f. multivariate regression result of hypertension controlled

Hypertension controlled	Odds Ratio	Std.Err	Z	P>|Z|	95% conf. Interval	
**Mental health disorder**						
**Yes**	1.06	0.11	0.54	0.589	0.87	1.29

**Year**						
**2015**	1.15	0.10	1.57	0.117	0.97	1.36
**No. of physical comorbidities**						
**One**	1.01	0.13	0.09	0.929	0.79	1.30
**Two**	1.06	0.14	0.44	0.661	0.81	1.38
**Three**	1.40	0.22	2.17	0.030	1.03	1.89
**Four and more**	1.16	0.19	0.90	0.367	0.84	1.60
**Gender**						
**Female**	0.95	0.09	-0.52	0.601	0.79	1.15
**Education level**						
**Middle or high school**	1.03	0.12	0.29	0.775	0.82	1.30
**College or above**	1.09	0.27	0.36	0.716	0.67	1.77
**Marital status**						
**Not married**	0.82	0.11	-1.39	0.165	0.63	1.08
**Age**						
**55-64**	0.96	0.12	-0.29	0.769	0.76	1.23
**65-74**	0.73	0.10	-2.21	0.027	0.56	0.97
**75+**	0.48	0.10	-3.69	0.000	0.33	0.71
**Consumption level**						
**Q2**	1.19	0.16	1.34	0.181	0.92	1.54
**Q3**	1.45	0.20	2.71	0.007	1.11	1.89
**Q4**	1.19	0.18	1.20	0.232	0.89	1.59
**Q5 (most affluent)**	1.35	0.21	1.95	0.051	1.00	1.84
**Insurance**						
**UEMI**	1.51	0.49	1.25	0.210	0.79	2.86
**URMI**	1.76	0.61	1.63	0.103	0.89	3.49
**NCMI**	1.33	0.40	0.96	0.337	0.74	2.40
**Others**	0.90	0.35	-0.28	0.782	0.41	1.95
**Residency**						
**Urban**	1.26	0.25	1.16	0.247	0.85	1.87
**Migrate**	0.90	0.12	-0.80	0.423	0.69	1.17
**No. of outpatient visit**	1.03	0.03	0.98	0.325	0.97	1.09
**Region**						
**Central**	0.88	0.14	-0.78	0.436	0.65	1.20
**Western**	1.03	0.13	0.21	0.834	0.80	1.31
**North-east**	1.06	0.36	0.16	0.870	0.54	2.07
**_cons**	0.46	0.16	-2.22	0.026	0.23	0.91

Note: UEMI=Urban Employee Medical Insurance; URMI=Urban Resident Medical Insurance; NCMI= New Cooperative Medical Insurance; Std.Err= standard error; conf.Interval= confidence interval.

Table 3a. univariate regression result of diabetes awareness

diabetes awareness	Odds Ratio	Std.Err	Z	P>|Z|	95% conf. Interval	

**Mental health disorder**						
**yes**	2.00	0.39	3.52	0.000	1.36	2.94
**_cons**	1.14	0.14	1.04	0.298	0.89	1.45

Note: Std.Err= standard error; conf.Interval= confidence interval.

Table 3b. multivariate regression result of diabetes awareness

diabetes awareness	Odds Ratio	Std.Err	Z	P>|Z|	95% conf. Interval	
**Mental health disorder**						
**Yes**	1.29	0.34	0.95	0.341	0.77	2.16
**Year**						
**2015**	13.84	4.26	8.52	0.000	7.56	25.31
**No. of physical comorbidities**						
**One**	2.33	0.81	2.43	0.015	1.18	4.60
**Two**	5.63	2.15	4.52	0.000	2.66	11.92
**Three**	18.62	8.72	6.24	0.000	7.44	46.65
**Four and more**	54.27	30.97	7.00	0.000	17.73	166.11
**Gender**						
**Female**	2.64	0.70	3.66	0.000	1.57	4.44
**Education level**						
**Middle or high school**	1.25	0.38	0.73	0.463	0.69	2.27
**College or above**	2.11	1.30	1.21	0.226	0.63	7.04
**Marital status**						
**Not married**	0.67	0.26	-1.02	0.306	0.32	1.43
**Age**						
**55-64**	1.58	0.47	1.55	0.121	0.89	2.83
**65-74**	1.71	0.61	1.51	0.132	0.85	3.44
**75+**	1.13	0.58	0.24	0.808	0.41	3.11
**Consumption level**						
**Q2**	1.77	0.61	1.66	0.096	0.90	3.46
**Q3**	1.68	0.60	1.45	0.147	0.83	3.38
**Q4**	2.01	0.75	1.86	0.063	0.96	4.19
**Q5 (most affluent)**	2.51	1.01	2.29	0.022	1.14	5.52
**Insurance**						
**UEMI**	1.25	0.90	0.32	0.751	0.31	5.09
**URMI**	0.69	0.52	-0.49	0.626	0.16	3.06
**NCMI**	0.57	0.34	-0.95	0.344	0.17	1.84
**Others**	1.24	1.10	0.24	0.811	0.22	7.02
**Residency**						
**Urban**	2.51	1.28	1.81	0.071	0.93	6.82
**Migrate**	1.49	0.52	1.15	0.250	0.75	2.95
**No. of outpatient visit**	1.29	0.11	2.89	0.004	1.08	1.52
**Region**						
**Central**	0.46	0.20	-1.78	0.075	0.19	1.08

**Western**	0.49	0.16	-2.13	0.033	0.26	0.94
**North-east**	1.72	1.70	0.55	0.583	0.25	11.92
**_cons**	0.03	0.02	-4.34	0.000	0.005	0.14

Note: UEMI=Urban Employee Medical Insurance; URMI=Urban Resident Medical Insurance; NCMI= New Cooperative Medical Insurance; Std.Err= standard error; conf.Interval= confidence interval.

Table 3c. univariate regression result of diabetes treated

Diabetes treated	Odds Ratio	Std.Err	Z	P>|Z|	95% conf. Interval	
**Mental health disorder**						
**yes**	0.97	0.23	-0.14	0.888	0.60	1.55
**_cons**	4.44	0.98	6.74	0.000	2.88	6.85

Note: Std.Err= standard error; conf.Interval= confidence interval.

Table 3d. multivariate regression result of diabetes treated

Diabetes treated	Odds Ratio	Std.Err	Z	P>|Z|	95% conf. Interval	
**Mental health disorder**						
**Yes**	0.92	0.24	-0.31	0.759	0.55	1.54
**Year**						
**2015**	1.28	0.29	1.10	0.272	0.82	1.99
**No. of physical comorbidities**						
**One**	0.57	0.23	-1.39	0.163	0.25	1.26
**Two**	0.71	0.29	-0.83	0.404	0.32	1.58
**Three**	0.89	0.37	-0.28	0.781	0.39	2.01
**Four and more**	0.82	0.34	-0.47	0.636	0.36	1.87
**Gender**						
**Female**	0.80	0.21	-0.84	0.398	0.48	1.34
**Education level**						
**Middle or high school**	0.65	0.20	-1.38	0.166	0.36	1.19
**College or above**	0.70	0.40	-0.62	0.533	0.23	2.16
**Marital status**						
**Not married**	1.08	0.42	0.20	0.839	0.50	2.33
**Age**						
**55-64**	1.56	0.49	1.43	0.152	0.85	2.87
**65-74**	1.15	0.42	0.38	0.704	0.56	2.34
**75+**	1.02	0.53	0.04	0.972	0.36	2.85
**Consumption level**						
**Q2**	0.90	0.33	-0.28	0.777	0.45	1.83
**Q3**	0.76	0.28	-0.75	0.452	0.37	1.55
**Q4**	1.57	0.61	1.16	0.248	0.73	3.38
**Q5 (most affluent)**	0.91	0.36	-0.25	0.801	0.42	1.96
**Insurance**						

**UEMI**	0.59	0.47	-0.66	0.510	0.12	2.82
**URMI**	0.46	0.38	-0.94	0.348	0.09	2.33
**NCMI**	0.47	0.34	-1.06	0.291	0.11	1.92
**Others**	2.01	2.07	0.68	0.497	0.27	15.07
**Residency**						
**Urban**	1.51	0.75	0.83	0.406	0.57	4.01
**Migrate**	1.29	0.44	0.75	0.452	0.66	2.53
**No. of outpatient visit**	1.07	0.08	1.01	0.315	0.93	1.24
**Region**						
**Central**	1.30	0.54	0.63	0.526	0.58	2.94
**Western**	1.01	0.30	0.03	0.977	0.56	1.82
**North-east**	0.98	0.80	-0.03	0.977	0.20	4.89
**_cons**	8.12	7.28	2.34	0.019	1.40	47.07

Note: UEMI=Urban Employee Medical Insurance; URMI=Urban Resident Medical Insurance; NCMI= New Cooperative Medical Insurance; Std.Err= standard error; conf.Interval= confidence interval

Table 3e. univariate regression result of diabetes controlled

diabetes controlled	Odds Ratio	Std.Err	Z	P>|Z|	95% conf. Interval	
**Mental health disorder**						
**yes**	1.69	0.42	2.09	0.037	1.03	2.76
**_cons**	0.45	0.08	-4.52	0.000	0.31	0.63

Note: Std.Err= standard error; conf.Interval= confidence interval.

Table 3f. multivariate regression result of diabetes controlled

diabetes controlled	Odds Ratio	Std.Err	Z	P>|Z|	95% conf. Interval	
**Mental health disorder**						
**Yes**	1.26	0.32	0.90	0.367	0.76	2.09
**Year**						
**2015**	1.86	0.45	2.56	0.010	1.16	3.00
**No. of physical comorbidities**						
**One**	1.56	0.64	1.07	0.282	0.69	3.50
**Two**	2.80	1.18	2.46	0.014	1.23	6.38
**Three**	4.08	1.79	3.20	0.001	1.73	9.63
**Four and more**	4.22	1.85	3.28	0.001	1.79	9.96
**Gender**						
**Female**	0.91	0.22	-0.40	0.690	0.56	1.47
**Education level**						
**Middle or high school**	1.50	0.44	1.37	0.171	0.84	2.68
**College or above**	1.06	0.57	0.12	0.908	0.37	3.02
**Marital status**						
**Not married**	1.19	0.46	0.46	0.645	0.56	2.52

**Age**						
**55-64**	0.63	0.19	-1.51	0.132	0.34	1.15
**65-74**	0.97	0.35	-0.07	0.941	0.48	1.97
**75+**	0.98	0.50	-0.03	0.973	0.36	2.67
**Consumption level**						
**Q2**	0.71	0.26	-0.95	0.344	0.35	1.45
**Q3**	0.57	0.21	-1.50	0.134	0.27	1.19
**Q4**	0.91	0.34	-0.26	0.797	0.44	1.88
**Q5 (most affluent)**	1.02	0.40	0.04	0.964	0.47	2.21
**Insurance**						
**UEMI**	0.50	0.37	-0.95	0.343	0.12	2.09
**URMI**	1.18	0.87	0.23	0.822	0.28	5.02
**NCMI**	0.70	0.47	0.53	0.595	0.19	2.62
**Others**	0.67	0.59	-0.46	0.648	0.12	3.71
**Residency**						
**Urban**	0.54	0.25	-1.35	0.177	0.22	1.33
**Migrate**	0.34	0.12	-3.00	0.003	0.17	0.69
**No. of outpatient visit**	1.01	0.06	0.21	0.835	0.90	1.14
**Region**						
**Central**	2.39	0.97	2.15	0.032	1.08	5.31
**Western**	1.32	0.39	0.94	0.348	0.74	2.34
**North-east**	2.32	1.75	1.11	0.265	0.53	10.18
**_cons**	0.28	0.23	-1.53	0.127	0.06	1.43

Note: UEMI=Urban Employee Medical Insurance; URMI=Urban Resident Medical Insurance; NCMI= New Cooperative Medical Insurance; Std.Err= standard error; conf.Interval= confidence interval.

Table 4a. univariate regression result of dyslipidemia awareness

Dyslipidemia awareness	Odds Ratio	Std.Err	Z	P>|Z|	95% conf. Interval	
**Mental health disorder**						
**yes**	2.88	0.38	7.90	0.000	2.21	3.74
**_cons**	0.10	0.01	-15.60	0.000	0.08	0.14

Note: Std.Err= standard error; conf.Interval= confidence interval.

Table 4b. multivariate regression result of dyslipidemia awareness

Dyslipidemia awareness	Odds Ratio	Std.Err	Z	P>|Z|	95% conf. Interval	
**Mental health disorder**						
**Yes**	2.09	0.33	4.61	0.000	1.53	2.86
**year**						
**2015**	5.24	0.74	11.69	0.000	3.97	6.92
**No. of physical comorbidities**						
**One**	4.77	1.01	7.36	0.000	3.15	7.23
**Two**	16.78	4.16	11.37	0.000	10.32	27.29

**Three**	50.82	15.28	13.07	0.000	28.19	91.60
**Four or more**	135.67	46.17	14.43	0.000	69.63	264.34
**Gender**						
**Female**	1.33	0.20	1.90	0.057	0.99	1.78
**Education level**						
**Middle or high school**	1.87	0.33	3.51	0.000	1.32	2.65
**College or above**	5.52	2.06	4.58	0.000	2.66	11.46
**Marital status**						
**Not married**	0.89	0.21	-0.50	0.620	0.56	1.41
**Age**						
**55-64**	1.41	0.23	2.04	0.041	1.01	1.95
**65-74**	1.37	0.28	1.52	0.128	0.91	2.04
**75+**	0.68	0.22	-1.18	0.240	0.36	1.29
**Consumption level**						
**Q2**	1.15	0.23	0.69	0.488	0.78	1.70
**Q3**	0.99	0.21	-0.02	0.980	0.66	1.50
**Q4**	1.37	0.30	1.45	0.146	0.90	2.10
**Q5 (most affluent)**	1.65	0.38	2.19	0.029	1.05	2.60
**Insurance**						
**UEMI**	5.32	2.61	3.40	0.001	2.03	13.94
**URMI**	2.54	1.32	1.79	0.073	0.92	7.01
**NCMI**	2.00	0.88	1.58	0.114	0.85	4.75
**Others**	2.57	1.49	1.63	0.104	0.82	8.00
**Residency**						
**Urban**	2.25	0.72	2.53	0.011	1.20	4.23
**Migrate**	1.27	0.29	1.04	0.297	0.81	1.99
**No. of outpatient visit**	1.10	0.04	2.32	0.020	1.01	1.19
**Region**						
**Central**	0.47	0.14	-2.53	0.011	0.26	0.84
**Western**	0.82	0.19	-0.86	0.387	0.52	1.29
**North-east**	0.76	0.48	-0.44	0.661	0.22	2.62
**_cons**	0.0006	0.0004	-11.59	0.000	0.0002	0.002

Note: UEMI=Urban Employee Medical Insurance; URMI=Urban Resident Medical Insurance; NCMI= New Cooperative Medical Insurance; Std.Err= standard error; conf.Interval= confidence interval.

Table 4c. univariate regression result of dyslipidemia treated

Dyslipidemia treated	Odds Ratio	Std.Err	Z	P>|Z|	95% conf. Interval	
**Mental health disorder**						
**yes**	1.74	0.23	4.10	0.000	1.33	2.26
**_cons**	1.20	0.10	2.27	0.024	1.02	1.40

Note: Std.Err= standard error; conf.Interval= confidence interval.

Table 4d. multivariate regression result of dyslipidemia treated

Dyslipidemia treated	Odds Ratio	Std.Err	Z	P>|Z|	95% conf. Interval	
**Mental health disorder**						
**Yes**	1.28	0.17	1.85	0.065	0.98	1.68
**year**						
**2015**	0.82	0.10	-1.60	0.109	0.65	1.04
**No. of physical comorbidities**						
**One**	1.95	0.44	2.96	0.003	1.25	3.03
**Two**	2.34	0.52	3.79	0.000	1.51	3.62
**Three**	3.17	0.77	4.75	0.000	1.97	5.11
**Four or more**	4.49	1.14	5.90	0.000	2.72	7.39
**Gender**						
**Female**	0.89	0.11	-0.95	0.341	0.69	1.14
**Education level**						
**Middle or high school**	0.76	0.11	-1.89	0.059	0.57	1.01
**College or above**	0.86	0.22	-0.59	0.553	0.51	1.43
**Marital status**						
**Not married**	0.99	0.20	-0.03	0.979	0.67	1.47
**Age**						
**55-64**	1.33	0.20	1.88	0.060	0.99	1.78
**65-74**	1.04	0.18	0.22	0.828	0.74	1.46
**75+**	0.88	0.26	-0.42	0.673	0.50	1.56
**Consumption level**						
**Q2**	1.11	0.21	0.55	0.580	0.77	1.60
**Q3**	1.05	0.20	0.26	0.798	0.72	1.54
**Q4**	1.03	0.20	0.16	0.871	0.70	1.51
**Q5 (most affluent)**	1.06	0.21	0.28	0.780	0.71	1.58
**Insurance**						
**UEMI**	0.70	0.33	-0.78	0.438	0.28	1.74
**URMI**	0.60	0.30	-1.03	0.303	0.23	1.58
**NCMI**	0.93	0.42	-0.15	0.878	0.38	2.26
**Others**	0.80	0.45	-0.39	0.695	0.27	2.39
**Residency**						
**Urban**	0.91	0.22	-0.38	0.707	0.57	1.46
**Migrate**	1.20	0.20	1.05	0.295	0.86	1.67
**No. of outpatient visit**	1.10	0.04	2.50	0.012	1.02	1.18
**Region**						
**Central**	1.19	0.23	0.87	0.382	0.81	1.74
**Western**	0.98	0.15	-0.17	0.869	0.72	1.31
**North-east**	0.68	0.28	-0.94	0.346	0.31	1.51
**_cons**	0.65	0.34	-0.84	0.401	0.23	1.79

Note: UEMI=Urban Employee Medical Insurance; URMI=Urban Resident Medical Insurance; NCMI= New Cooperative Medical Insurance; Std.Err= standard error; conf.Interval= confidence interval.

Table 4e. univariate regression result of dyslipidemia controlled

Dyslipidemia controlled	Odds Ratio	Std.Err	Z	P>|Z|	95% conf. Interval	
**Mental health disorder**						
**yes**	1.07	0.22	0.35	0.729	0.72	1.60
**_cons**	0.87	0.11	-1.12	0.265	0.68	1.11

Note: Std.Err= standard error; conf.Interval= confidence interval.

Table 4f. multivariate regression result of dyslipidemia controlled

Dyslipidemia controlled	Odds Ratio	Std.Err	Z	P>|Z|	95% conf. Interval	
**Mental health disorder**						
**Yes**	0.93	0.21	-0.30	0.762	0.60	1.45
**year**						
**2015**	1.61	0.32	2.38	0.017	1.09	2.39
**No. of physical comorbidities**						
**One**	1.00	0.44	0.00	1.000	0.42	2.39
**Two**	0.96	0.42	-0.10	0.921	0.41	2.25
**Three**	1.05	0.47	0.11	0.913	0.44	2.53
**Four and more**	0.75	0.34	-0.64	0.524	0.31	1.81
**Gender**						
**Female**	1.47	0.33	1.71	0.087	0.95	2.27
**Education level**						
**Middle or high school**	1.18	0.31	0.65	0.514	0.71	1.97
**College or above**	1.13	0.54	0.25	0.806	0.44	2.90
**Marital status**						
**Not married**	1.09	0.37	0.26	0.795	0.56	2.13
**Age**						
**55-64**	1.11	0.29	0.40	0.690	0.67	1.84
**64-74**	1.39	0.44	1.04	0.300	0.75	2.57
**75+**	1.21	0.64	0.36	0.722	0.43	3.40
**Consumption level**						
**Q2**	0.64	0.21	-1.39	0.163	0.34	1.20
**Q3**	0.52	0.17	-1.96	0.050	0.27	1.00
**Q4**	0.75	0.26	-0.84	0.402	0.39	1.46
**Q5**	0.45	0.16	-2.22	0.027	0.22	0.91
**Insurance**						
**UEMI**	2.76	2.26	1.24	0.214	0.56	13.69
**URMI**	1.47	1.27	0.45	0.655	0.27	7.97
**NCMI**	2.91	2.35	1.32	0.185	0.60	14.15
**Others**	2.32	2.26	0.86	0.388	0.34	15.62
**Residency**						

Urban	0.65	0.26	-1.08	0.280	0.30	1.42
Migrate	0.68	0.19	-1.40	0.161	0.40	1.16
**No. of outpatient visit**	1.00	0.05	0.06	0.955	0.91	1.10
**Region**						
Central	1.01	0.30	0.04	0.970	0.56	1.82
Western	1.18	0.29	0.68	0.499	0.73	1.89
North-east	0.84	0.53	-0.28	0.779	0.24	2.93
**_cons**	0.31	0.30	-1.23	0.219	0.05	2.01

Note: UEMI=Urban Employee Medical Insurance; URMI=Urban Resident Medical Insurance; NCMI= New Cooperative Medical Insurance; Std.Err= standard error; conf.Interval= confidence interval.

Table 5a. univariate regression result of kidney disease awareness

Kidney disease awareness	Odds Ratio	Std.Err	Z	P>|Z|	95% conf. Interval	
**Mental health disorder**						
yes	4.14	0.71	8.23	0.000	2.95	5.81
**_cons**	0.003	0.0009	-18.23	0.000	0.001	0.005

Note: Std.Err= standard error; conf.Interval= confidence interval.

Table 5b. multivariate regression result of kidney disease awareness

Kidney disease awareness	Odds Ratio	Std.Err	Z	P>|Z|	95% conf. Interval	
**Mental health disorder**						
Yes	3.02	0.53	6.34	0.000	2.15	4.25
**Year**						
2015	0.90	0.12	-0.79	0.430	0.69	1.17
**No. of physical comorbidities**						
One	2.88	0.66	4.61	0.000	1.84	4.50
Two	6.78	1.67	7.79	0.000	4.19	10.99
Three	17.73	5.26	9.70	0.000	9.92	31.70
Four or more	78.06	25.49	13.35	0.000	41.16	148.04
**Gender**						
Female	0.31	0.06	-6.57	0.000	0.22	0.44
**Education level**						
Middle or high school	1.14	0.24	0.62	0.534	0.76	1.71
College or above	0.79	0.37	-0.50	0.616	0.31	1.99
**Marital status**						
Not married	0.60	0.16	-1.94	0.053	0.36	1.01
**Age**						
55-64	0.56	0.11	-2.94	0.003	0.38	0.82
65-74	0.33	0.08	-4.62	0.000	0.21	0.53
75+	0.26	0.09	-3.95	0.000	0.13	0.50
**Consumption level**						

**Q2**	1.21	0.27	0.84	0.399	0.78	1.87
**Q3**	1.07	0.24	0.28	0.780	0.68	1.67
**Q4**	0.99	0.24	-0.06	0.953	0.61	1.59
**Q5 (most affluent)**	1.63	0.42	1.89	0.058	0.98	2.69
**Insurance**						
**UEMI**	2.97	1.74	1.86	0.063	0.94	9.38
**URMI**	5.37	3.33	2.71	0.007	1.59	18.11
**NCMI**	2.36	1.21	1.67	0.096	0.86	6.46
**Others**	2.77	1.89	1.50	0.134	0.73	10.53
**Residency**						
**Urban**	0.56	0.19	-1.67	0.095	0.29	1.11
**Migrate**	0.84	0.18	-0.85	0.393	0.55	1.26
**No. of outpatient visit**	1.17	0.05	3.38	0.001	1.07	1.28
**Region**						
**Central**	2.00	0.62	2.21	0.027	1.08	3.68
**Western**	0.92	0.23	-0.35	0.727	0.56	1.50
**North-east**	2.30	1.29	1.49	0.136	0.77	6.88
**_cons**	0.001	0.0005	-10.86	0.000	0.0002	0.003

Note: UEMI=Urban Employee Medical Insurance; URMI=Urban Resident Medical Insurance; NCMI= New Cooperative Medical Insurance; Std.Err= standard error; conf.Interval= confidence interval.

Table 5c. univariate regression result of kidney disease treated

Kidney disease treated	Odds Ratio	Std.Err	Z	P>|Z|	95% conf. Interval	
**Mental health disorder**						
**yes**	1.50	0.24	2.50	0.012	1.09	2.05
**_cons**	0.93	0.10	-0.72	0.474	0.76	1.14

Note: Std.Err= standard error; conf.Interval= confidence interval.

Table 5d. multivariate regression result of kidney disease treated

Kidney disease treated	Odds Ratio	Std.Err	Z	P>|Z|	95% conf. Interval	
**Mental health disorder**						
**Yes**	1.34	0.22	1.72	0.086	0.96	1.86
**Year**						
**2015**	0.81	0.12	-1.35	0.177	0.60	1.10
**No. of physical comorbidities**						
**One**	1.57	0.44	1.60	0.109	0.90	2.74
**Two**	1.65	0.47	1.77	0.076	0.95	2.87
**Three**	1.41	0.42	1.13	0.257	0.78	2.54
**Four or more**	1.85	0.54	2.11	0.035	1.05	3.26
**Gender**						
**Female**	0.87	0.14	-0.87	0.382	0.63	1.19

**Education level**						
**Middle or high school**	1.27	0.24	1.26	0.206	0.88	1.84
**College or above**	1.41	0.63	0.77	0.443	0.59	3.37
**Marital status**						
**Not married**	1.46	0.38	1.45	0.146	0.88	2.42
**Age**						
**55-64**	1.03	0.19	0.16	0.873	0.71	1.49
**65-74**	0.97	0.22	-0.12	0.902	0.62	1.52
**75+**	0.86	0.30	-0.44	0.661	0.44	1.69
**Consumption level**						
**Q2**	1.25	0.29	0.97	0.330	0.80	1.97
**Q3**	1.04	0.25	0.17	0.866	0.65	1.66
**Q4**	0.99	0.25	-0.04	0.965	0.60	1.62
**Q5 (most affluent)**	1.55	0.40	1.70	0.090	0.93	2.56
**Insurance**						
**UEMI**	1.27	0.77	0.39	0.697	0.39	4.16
**URMI**	2.40	1.55	1.36	0.175	0.68	8.54
**NCMI**	1.88	1.04	1.14	0.253	0.64	5.53
**others**	2.30	1.65	1.16	0.248	0.56	9.42
**Residency**						
**Urban**	0.78	0.26	-0.75	0.453	0.41	1.49
**Migrate**	1.36	0.28	1.48	0.139	0.91	2.03
**No. of outpatient visit**	1.04	0.04	1.00	0.319	0.96	1.12
**Region**						
**Central**	2.51	0.60	3.86	0.000	1.57	4.00
**Western**	1.51	0.30	2.10	0.036	1.03	2.22
**North-east**	1.15	0.52	0.32	0.751	0.48	2.79
**_cons**	0.20	0.13	-2.46	0.014	0.06	0.72

Note: UEMI=Urban Employee Medical Insurance; URMI=Urban Resident Medical Insurance; NCMI= New Cooperative Medical Insurance; Std.Err= standard error; conf.Interval= confidence interval.

Table 5e. univariate regression result of kidney disease controlled

Kidney disease controlled	Odds Ratio	Std.Err	Z	P>|Z|	95% conf. Interval	
**Mental health disorder**						
**yes**	1.21	0.36	0.64	0.523	0.67	2.18
**_cons**	0.60	0.14	-2.17	0.030	0.38	0.95

Note: Std.Err= standard error; conf.Interval= confidence interval.

Table 5f. multivariate regression result of kidney disease controlled

Kidney disease controlled	Odds Ratio	Std.Err	Z	P>|Z|	95% conf. Interval	
**Mental health disorder**						

**Yes**	1.03	0.32	0.10	0.918	0.57	1.88
**Year**						
**2015**	0.85	0.23	-0.58	0.559	0.50	1.45
**No. of physical comorbidities**						
**One**	0.77	0.42	-0.48	0.628	0.27	2.22
**Two**	0.63	0.34	-0.85	0.398	0.22	1.83
**Three**	1.11	0.64	0.18	0.856	0.36	3.44
**Four or more**	1.03	0.56	0.05	0.963	0.35	3.00
**Gender**						
**Female**	1.13	0.34	0.42	0.674	0.63	2.04
**Education level**						
**Middle or high school**	0.95	0.33	-0.16	0.874	0.48	1.86
**College or above**	2.86	2.53	1.18	0.237	0.50	16.24
**Marital status**						
**Not married**	1.67	0.77	1.11	0.265	0.68	4.11
**Age**						
**55-64**	0.45	0.17	-2.13	0.033	0.22	0.94
**64-74**	0.20	0.10	-3.22	0.001	0.08	0.53
**75+**	0.07	0.06	-3.20	0.001	0.01	0.35
**Consumption level**						
**Q2**	0.58	0.26	-1.21	0.226	0.24	1.40
**Q3**	0.77	0.34	-0.59	0.555	0.32	1.84
**Q4**	0.52	0.26	-1.30	0.194	0.20	1.39
**Q5 (most affluent)**	0.40	0.20	-1.79	0.073	0.15	1.09
**Insurance**						
**UEMI**	1.42	1.72	0.29	0.772	0.13	15.29
**URMI**	3.02	3.81	0.88	0.379	0.26	35.67
**NCMI**	1.64	1.71	0.48	0.633	0.21	12.70
**others**	2.03	2.79	0.52	0.605	0.14	29.91
**Residency**						
**Urban**	0.79	0.50	-0.37	0.711	0.23	2.76
**Migrate**	0.90	0.34	-0.28	0.779	0.42	1.90
**No. of outpatient visit**	0.97	0.08	-0.41	0.681	0.83	1.13
**Region**						
**Central**	1.31	0.59	0.60	0.547	0.54	3.18
**Western**	0.82	0.33	-0.50	0.616	0.37	1.81
**North-east**	1.20	1.12	0.20	0.845	0.19	7.42
**_cons**	1.88	2.30	0.51	0.607	0.17	20.66

Note: UEMI=Urban Employee Medical Insurance; URMI=Urban Resident Medical Insurance; NCMI= New Cooperative Medical Insurance; Std.Err= standard error; conf.Interval= confidence interval.
